# Imaging characteristics of nodal metastases in paraganglioma, ameloblastoma and olfactory neuroblastoma: case reports and literature review

**DOI:** 10.1259/bjrcr.20150096

**Published:** 2016-07-28

**Authors:** Smita Deb, Tim Anton Iseli, Timothy Wong, Pramit M Phal

**Affiliations:** ^1^Department of Surgery, Royal Melbourne Hospital, University of Melbourne, Melbourne, VIC, Australia; ^2^Department of Radiology, Royal Melbourne Hospital, University of Melbourne, Melbourne, VIC, Australia

## Abstract

Paraganglioma, ameloblastoma and olfactory neuroblastoma are uncommon primary head and neck tumours. When nodal metastases from these tumours occur, they may present later than and with different imaging characteristics compared with squamous cell carcinoma (SCC), demonstrating appearances similar to the primary tumour type rather than features typical of metastatic nodal SCC. We present three cases in which imaging characteristics of nodal metastases in paraganglioma, ameloblastoma and olfactory neuroblastoma mimicked the primary tumour and discuss their implications for clinicoradiological follow-up.

## Background

Squamous cell carcinoma (SCC) accounts for 90% of all head and neck tumours and nodal metastases occur frequently.^1,2^ These metastases have typical features on imaging, including enlarged size (>10 mm), rounded shape, perinodal stranding suggesting extracapsular spread, conglomerated nodes and central necrosis.^3^ Less common head and neck primary malignancies may also result in nodal metastases that have different imaging characteristics on CT and MRI, often mimicking the primary tumour.^4,5^ We present three cases of unusual head and neck primary tumoursand discuss the imaging characteristics of their nodal metastases: paraganglioma (PG), accounting for only 0.6% of all head and neck tumours; ameloblastoma, which makes up 1% of all oral cavity tumours; and olfactory neuroblastoma, which comprises 2% of all sinonasal cavity tumours.^5–9^

## Case 1—primary malignant vagal paraganglioma with nodal metastasis

**Figure 1.**
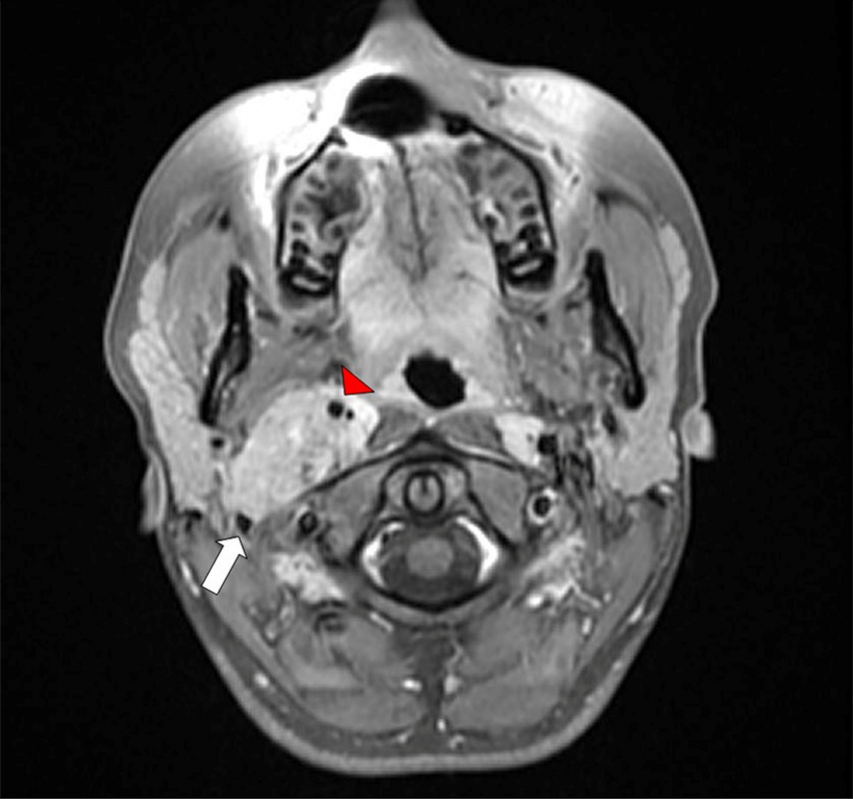
Axial *T*_1_ weighted MRI with contrast and fat suppression. Displacement of the internal carotid artery anteriorly (red arrow head) with the internal jugular vein pushed posteriorly (white arrow).

A 19-year-old female presented with sore throat, right-sided jaw pain and a right neck mass that had been present for the past 4 months. A neck ultrasound scan showed a markedly vascular mass in relation to the right carotid sheath. MRI showed an enhancing mass in the right carotid space near the bifurcation, extending superiorly to within 1 cm of the skull base. Characteristic splaying of the carotid artery and internal jugular vein (Figure 1), as well as typical contrast enhancement with flow voids suggested a vagal paraganglioma (VPG) with a less likely differential diagnosis of schwannoma. There were also multiple enlarged jugular chain and lateral retropharyngeal lymph nodes, which were felt likely to be reactive given the patient’s young age. Urinary catecholamines were in the normal range and MRI of the abdomen demonstrated no adrenal or extra-adrenal PG.

**Figure 2.**
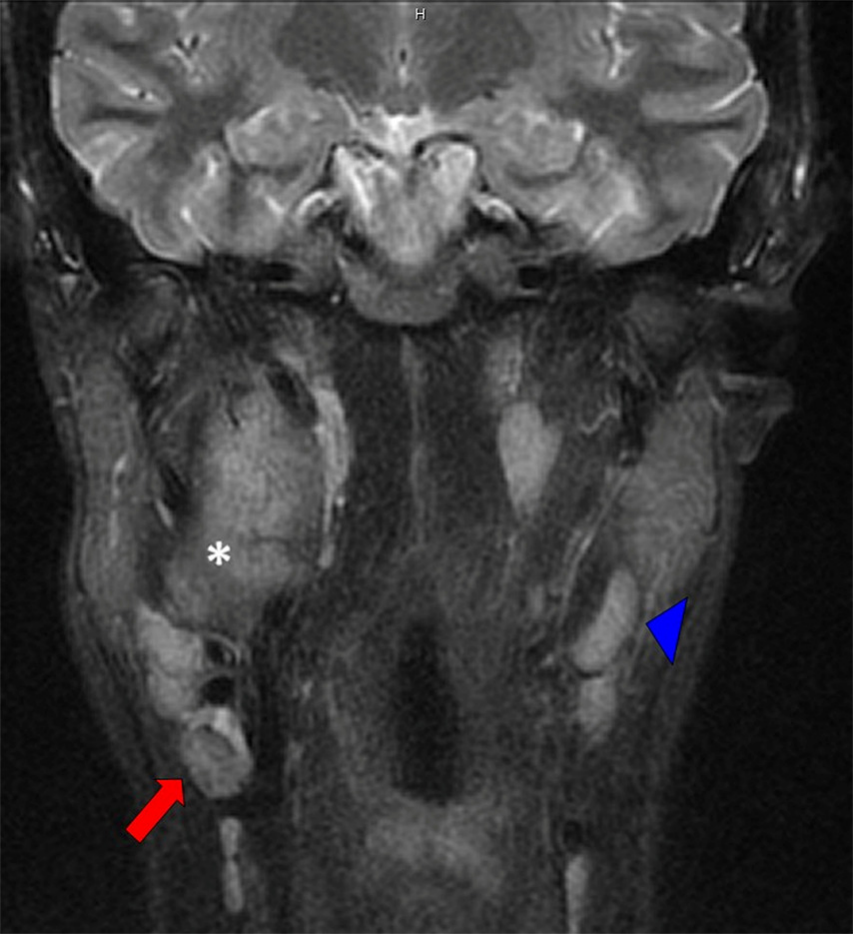
Coronal MRI short tau inversion-recovery sequence (STIR) demonstrating a right-sided carotid space mass of intermediate low signal (white asterisk) with an abnormal lymph node (red arrow) showing signal intensity similar to the mass. A contralateral normal lymph node is shown for comparison (blue arrowhead).

The lesion was approached surgically *via* a cervical incision and abnormal hypervascular solid lymph nodes were apparent in levels 2 and 3. Frozen section showed paraganglioma cells in the lymph nodes, confirming a malignant tumour. In retrospect, these nodes had similar signals and enhancement as the primary tumour (Figure 2). Resection of the malignant tumour was performed, which included sacrifice of cranial nerves X and XII. These were reconstructed with ansa cervicalis nerve transfer onto the recurrent laryngeal nerve for vocal cord tone and greater auricular nerve cable graft to the XII defect.

Formal histopathological examination demonstrated a malignant VPG involving 4 out of 13 level 2 and 3 cervical lymph nodes, and involved surgical margins at the skull base. The patient subsequently underwent post-operative radiotherapy and was referred for genetic testing and family counselling.

## Case 2—recurrent ameloblastoma with nodal metastasis

**Figure 3.**
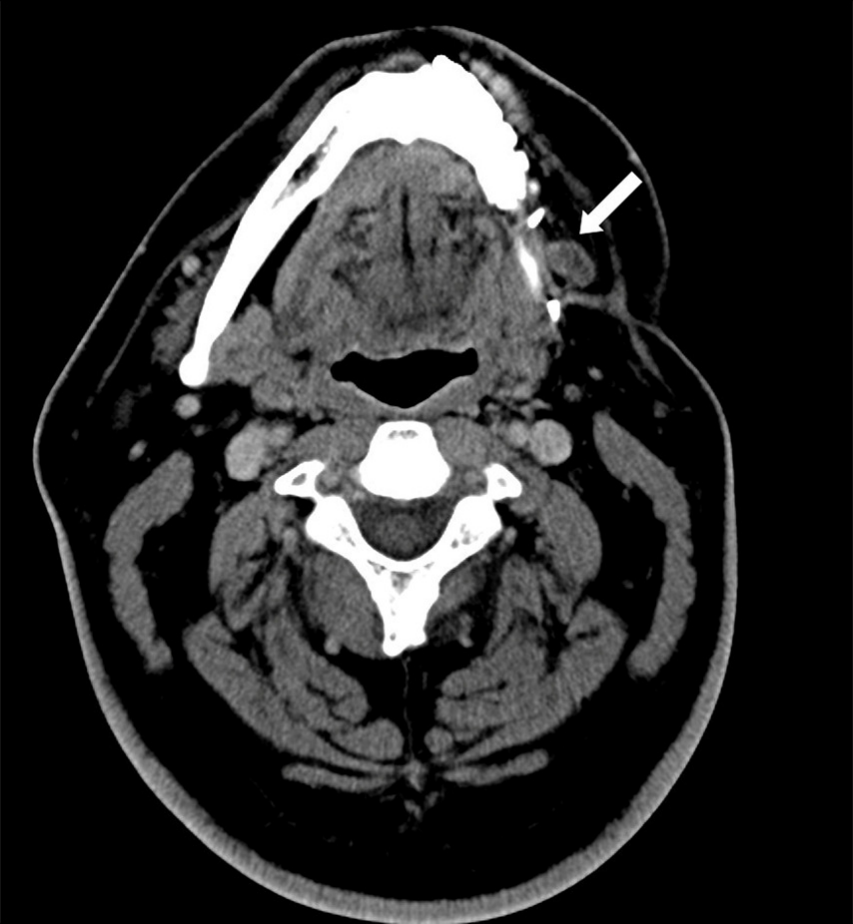
Axial CT scan showing a large pathological submandibular lymph node with heterogeneous internal density (white arrow).

A 54-year-old male presented with left jaw swelling with a background of a primary left mandibular ameloblastoma resected 4 years ago. CT suggested soft tissue recurrence lateral to the mandible as well as an enlarged left level 1b submandibular node with heterogeneous internal density (Figure 3). This was felt most likely to be reactive owing to the rarity of nodal metastases in ameloblastoma, and surgical planning was for excision of the local recurrence with primary closure of the neck skin.

The patient underwent excision of the soft tissue mass with en bloc resection of the node. Histological examination showed recurrent ameloblastoma in the soft tissue, with metastatic ameloblastoma within the submandibular lymph node. A second stage selective neck dissection of levels 1–3 was performed and 0/23 nodes contained malignancy. Radiation therapy was discussed with the patient but not undertaken. The patient is undergoing annual surveillance at our centre and has no evidence of recurrence 18 months post revision surgery.

## Case 3—rapidly advancing metastatic olfactory neuroblastoma (nodal, bone)

A 72-year-old female presented with right-sided neck swelling 1 year after craniofacial resection of a Hyams grade 2 olfactory neuroblastoma, which initially presented with 6 months of right-sided nasal obstruction, anosmia and facial swelling. The patient received adjuvant radiotherapy to the primary site.

**Figure 4.**
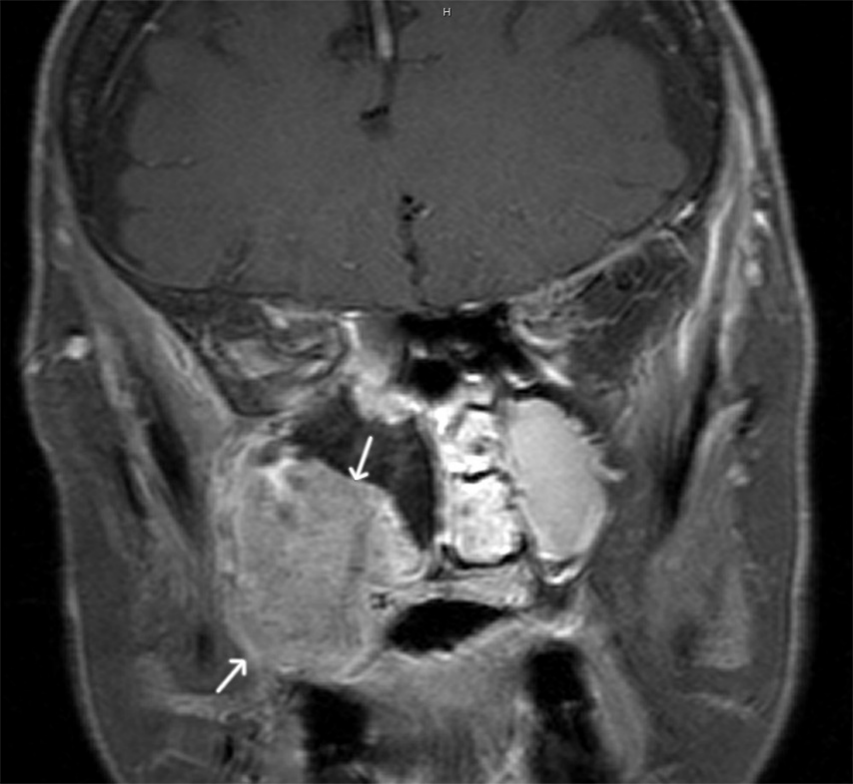
Coronal *T*_1_ weighted fat-saturated contras-enhanced image shows an enhancing olfactory neuroblastoma in the inferolateral wall of the right maxillary sinus (white arrows).

**Figure 5.**
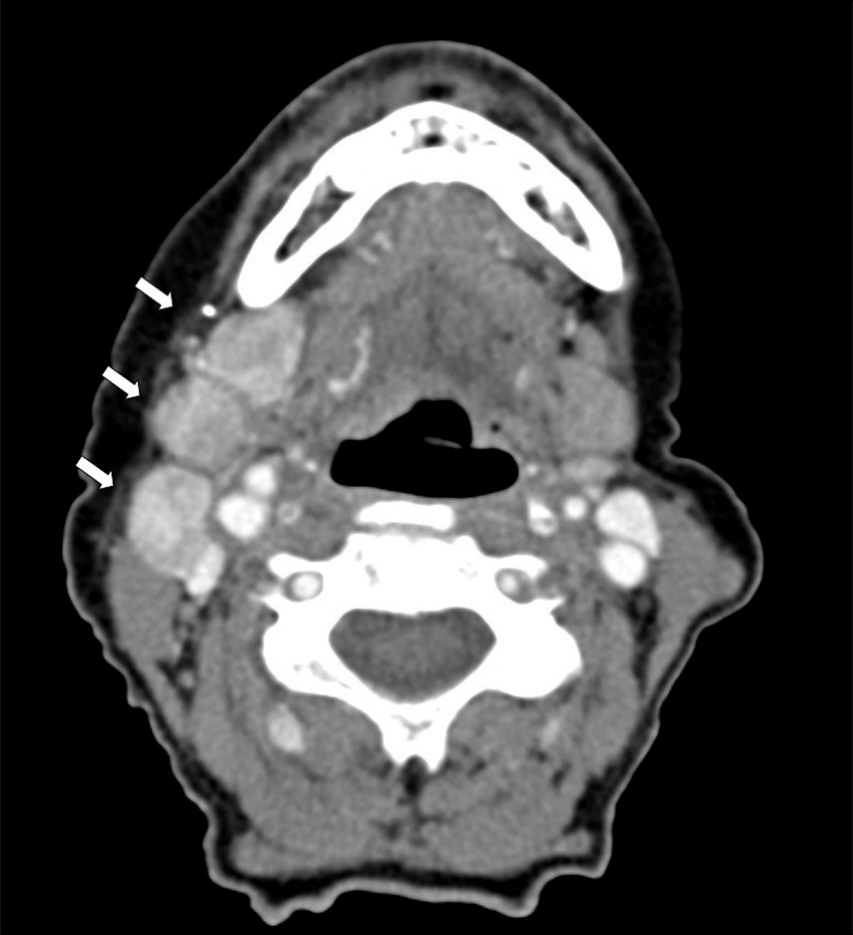
Axial CT with contrast demonstrating abnormal, hyperenhancing right levels 1B and 2 lymphadenopathy (white arrows).

Her initial MRI demonstrated the primary tumour as a large, destructive mass in the right maxillary sinus (Figure 4). Restaging CT scan of the neck at 12 months showed hyperenhancing cervical lymphadenopathy (Figure 5). Ultrasound examination showed hypervascular right levels 1B and 2 lymph nodes and was used to guide fine-needle aspiration, confirming metastatic olfactory neuroblastoma.

The patient went on to have a right-sided comprehensive neck dissection followed by radiotherapy to the right neck. Follow-up positron emission tomography (PET) at 3 months demonstrated T10 and left humerus metastases, from which the patient was asymptomatic. Local radiotherapy was provided to the T10 metastasis. However, the bony metastases progressed within the following year and the patient subsequently died.

## Discussion

Mucosal head and neck SCCs commonly present with metastatic cervical lymphadenopathy.^1^ CT and MRI of the neck are used to identify nodal metastasis, and ultrasound-guided node biopsy may confirm malignancy.^5^ There is limited literature addressing solely the role of PET/CT imaging in head and neck PG or ameloblastoma.^10^ However, PGs are avid for 6-^18^F-L-fluoro-L-3, 4-dihydroxyphenylalanine and recurrent PGs demonstrate high uptake with fluorine-18-fludeoxyglucose. Ameloblastomas and olfactory neuroblastomas may also show increased fluorine-18-fludeoxyglucose uptake. PET/CT scanning may be useful in detecting occult disease or metastatic lesions not otherwise demonstrated by MRI for all three tumour types discussed in this case series.^10–14^

Imaging characteristics on CT and MRI used for determining nodal malignancy in head and neck SCC include size criteria, change in nodal morphology from the normal reniform shape, loss of nodal fatty hilum, central necrosis, nodal conglomeration and extracapsular spread into adjacent nodes or other structures such as vessels.^3^ We illustrate three head and neck tumours that uncommonly present with nodal disease where imaging characteristics are less well described, namely PG, ameoblastoma and olfactory neuroblastoma. This case review also suggests that cervical metastases of these head and neck tumours often maintain imaging characteristics similar to those of the primary tumour.

The first case in this series demonstrated lymph node metastases in a VPG, 90% of which are benign tumours.^8^ The diagnosis of malignancy is often made retrospectively based on histology when PG cells are seen in lymph nodes. Central necrosis and extracapsular spread are hence unlikely to be seen in these tumours. Size criteria used for SCC nodal metastases (jugulodigastric nodes >15 mm, retropharyngeal nodes >8 mm, submandibular >5 mm and other nodes >10 mm) may have low specificity in young patients who commonly have larger reactive nodes.^3^ The key imaging finding that might raise suspicion for metastatic PG is low *T*_2_ signal and bright contrast enhancement similar to the primary mass.^4^

The second case demonstrated a very rare (<2%) lymph node metastasis from recurrent ameloblastoma of the mandible.^6,15^ Metastasizing ameloblastoma has occurred in 42 cases, of which cervical nodal metastasis made up only 10.^15^ Metastasizing ameloblastoma typically spreads to the lungs and then the cervical lymph nodes (Table 1).^6,7,15^ Metastatic risk factors include large primary, fast growth, presence of recurrence and treatment delays.^15^ Size criteria might again raise suspicion but the key feature in this case was the heterogeneous internal density of the enlarged node on CT , similar to the primary tumour but different from central necrosis in SCC, which is usually of low density centrally.

**Table 1.** Rate of metastasis by site^7,9,16^

	Paraganglioma (%)*^a^*	Ameloblastoma (%)*^a^*	Olfactory neuroblastoma (%)
Cervical lymph node	64	28	25
Lung	10	71	—
Bone	64	12	—
Liver	18	8	—
Intracranial	—	9	—
Other*^b^*	—	12	—

^*a*^These rates include cases in recent studies with multiple metastases.

*^b^*Includes spleen, kidneys, diaphragm, heart and skin for ameloblastomas.

Olfactory neuroblastomas metastasize to the neck in up to one-fourth of cases and approximately 8% of patients have cervical nodal metastases at the time of presentation.^9^ Nodal metastasis is common in late stage disease and indicates especially poor prognosis.^9,17^ Suspicion should be raised by enlarged nodes in typical locations (Table 2) and imaging features similar to the primary tumour.^5^ On CT and MRI, nodal metastases of olfactory neuroblastomas appear completely solid, without central necrosis, and show strong contrast enhancement with similar attenuation as the primary tumour. Enlarged nodes by size and even strongly enhancing nodes below normal size criteria should be considered suspicious for malignancy.^5^

**Table 2.** Rate of cervical nodal metastasis of olfactory neuroblastoma (%)*^a^*^5^

Cervical node level or site	
Level 1	93
Level 2	57
Level 3	50
Retropharyngeal	43

^*a*^Involvement of cervical nodes at levels 4 and 5 occurred in cases with widespread metastatic neck disease.

Typically, nodal metastases from SCC of the head and neck recur early after diagnosis of the primary tumour.^1^ In these three tumours, however, nodal metastases may occur many years after diagnosis of the primary tumour. The mean metastasis-free interval for PG is 8 years.^16,18^ Lymph nodes are the most common site of metastasis for both PG and olfactory neuroblastomas (Table 1).^16,18,19^ The mean disease-free interval for ameloblastoma, which includes recurrences, is 14 years.^6,7^ 40% of PG are inherited and these are more likely to present bilaterally, at a younger age and involve multiple sites.^8^ Thus, annual imaging surveillance has been advocated.^20^ Post-operative follow-up for ameloblastoma patients should be continued for up to25 years and include clinical examination and imaging as clinically indicated.^15,21^ Olfactory neuroblastoma recurrence rates are up to 50% and often within the first 2 years; therefore, one centre has suggested both contrast MRI and clinical examination every 4 months during this period with a step-down regime and lifelong follow-up.^18^

## Conclusions

Our case review illustrates nodal disease in three unusual head and neck tumours that uncommonly metastasize and their imaging characteristics on CT and MRI. Unlike head and neck SCC, nodal metastases in PG, ameloblastoma and olfactory neuroblastoma may not demonstrate typical imaging findings such as increased nodal size, central necrosis or loss of reniform shape, but instead may demonstrate nodal characteristics similar to the primary tumour such as avid contrast enhancement in the case of PG and olfactory neuroblastoma, and heterogeneous internal density on CT in the case of ameloblastoma. A high index of suspicion is required to detect nodal disease in these uncommon head and neck tumours, with clinicoradiological follow-up tailored to tumour type, including an awareness of the potential for late-presenting nodal disease.

## Learning points

1. Paraganglioma, ameloblastoma and olfactory neuroblastoma are uncommon head and neck tumours and often do not metastasize.
2. The metastatic lymph nodes for these tumours do not demonstrate typical CT or MRI findings in size, shape and morphology criteria applied to metastatic head and neck SCC. Instead, their nodal metastases can demonstrate findings similar to the primary tumour.
3. A high index of suspicion and regular clinico-radiological follow up to detect nodal disease in these three tumour types is advocated.
